# Community Tele-pal: A community-developed, culturally based palliative care tele-consult randomized controlled trial for African American and White Rural southern elders with a life-limiting illness

**DOI:** 10.1186/s13063-020-04567-w

**Published:** 2020-07-23

**Authors:** Kristen Allen Watts, Shena Gazaway, Emily Malone, Ronit Elk, Rodney Tucker, Susan McCammon, Michele Goldhagen, Jacob Graham, Veronica Tassin, Joshua Hauser, Sidney Rhoades, Marjorie Kagawa-Singer, Eric Wallace, James McElligott, Richard Kennedy, Marie Bakitas

**Affiliations:** 1 School of Medicine, Division of Gerontology, Geriatrics, and Palliative Care, University of Alabama at Birmingham, Birmingham, USA; 2 College of Nursing, Augusta University, Augusta, USA; 3 Center for Palliative and Supportive Care, University of Alabama at Birmingham, Birmingham, USA; 4 School of Medicine, Department of Otolaryngology, University of Alabama at Birmingham, Birmingham, USA; 5 Russell Medical Center, Alexander City, USA; 6 Forrest General Hospital, Hattiesburg, USA; 7 Highland Community Hospital, Picayune, USA; 8 Department of Medical Education at Northwestern University, Chicago, USA; 9 Aiken Regional Medical Center, Aiken, USA; 10 Fielding School of Public Health, Department of Community Health Sciences, University of California Los Angeles, Los Angeles, USA; 11 Department of Medicine, Division of Nephrology, University of Alabama at Birmingham, Birmingham, USA; 12 College of Medicine, The Medical University of South Carolina, Charleston, USA; 13 School of Nursing, University of Alabama at Birmingham, Birmingham, USA

**Keywords:** Palliative care, Tele-health, Tele-consultation, Rural hospitals, African Americans, Whites, Culturally based, Community-based participatory research

## Abstract

**Background:**

Patients living in rural areas experience a variety of unmet needs that result in healthcare disparities. The triple threat of rural geography, racial inequities, and older age hinders access to high-quality palliative care (PC) for a significant proportion of Americans. Rural patients with life-limiting illness are at risk of not receiving appropriate palliative care due to a limited specialty workforce, long distances to treatment centers, and limited PC clinical expertise. Although culture strongly influences people’s response to diagnosis, illness, and treatment preferences, culturally based care models are not currently available for most seriously ill rural patients and their family caregivers. The purpose of this randomized clinical trial (RCT) is to compare a culturally based tele-consult program (that was developed by and for the rural southern African American (AA) and White (W) population) to usual hospital care to determine the impact on symptom burden (primary outcome) and patient and care partner quality of life (QOL), care partner burden, and resource use post-discharge (secondary outcomes) in hospitalized AA and White older adults with a life-limiting illness.

**Methods:**

Community Tele-pal is a three-site RCT that will test the efficacy of a community-developed, culturally based PC tele-consult program for hospitalized rural AA and W older adults with life-limiting illnesses (*n* = 352) and a care partner. Half of the participants (*n* = 176) and a care partner (*n* = 176) will be randomized to receive the culturally based palliative care consult. The other half of the patient participants (*n* = 176) and care partners (*n* = 176) will receive usual hospital care appropriate to their illness.

**Discussion:**

This is the first community-developed, culturally based PC tele-consult program for rural southern AA and W populations. If effective, the tele-consult palliative program and methods will serve as a model for future culturally based PC programs that can reduce patients’ symptoms and care partner burden.

**Trial registration:**

ClinicalTrials.gov NCT03767517. Registered on 27 December 2018.

## Background

Inpatient palliative care (PC) consultations have identified unrecognized symptoms and unmet needs [1–3] and have been associated with lower ICU use [4, 5], fewer ICU deaths [6, 7], improved care processes, and higher rates of goals of care documentation [8, 9]. However, the triple threat of rural geography [10–13], racial inequities [14–16], and older age [17–19] hinders access to high-quality PC for a significant proportion of Americans.

Rural inpatients with limited access to PC expertise or lack of cultural tailoring when PC is available (uncertain acceptability) may experience in care disparities. In a state-by-state report card [20], PC access was ranked the lowest in the Southeastern United States, where a significant proportion of the population is rural and African American (AA). Rural patients with life-limiting illnesses are at very high risk of not receiving appropriate care due to a limited PC workforce, long distances to treatment centers, and limited PC clinical expertise [21, 22]. To address issues related to access in rural areas in this study, tele-health, the remote delivery of healthcare and sharing medical knowledge using telecommunication, will be used to provide specialty PC consultation via secure videoconferencing platforms to older AA and W patients with life-limiting illnesses and a care partner. Tele-health has been used as an avenue to improve care in remote areas for a variety of illnesses [23–36].

Acceptability of PC services is an equally consequential barrier in rural areas especially those with high proportions of minority patients, because research suggests that lack of cultural sensitivity may lead to mistrust of healthcare providers [27]. Culture is known to strongly influence people’s response to diagnosis, illness, and treatment preferences [28–30]; yet, culturally based care models are not currently available for most seriously ill rural patients and their care partners.

While other PC RCTs have measured the impact of tele-health [29, 37–40], a novel feature of this study’s intervention was the development by community stakeholders of a culturally based intervention, a key aspect of PC, since culture fundamentally shapes how individuals make meaning out of illness, suffering, and dying [41] and strongly influences their response to diagnosis, illness, and treatment preferences [41–43]. Community-based participatory research (CBPR) was the guiding method to develop this intervention, in which community members were equal partners. CBPR builds on community strengths; therefore, the community is integral to all phases of the research, including dissemination of findings [44]. Stakeholder input has been demonstrated to enhance both the quality and acceptability of interventions [45] as well as address health disparities, and results in demonstrable positive health outcomes [46]. In the community-developed, culturally based pilot study, we partnered with community stakeholders in creating the intervention, implementation, and testing for feasibility.

Phase I of the pilot took place in rural Beaufort, South Carolina (January 2013 to December 2013), where we assembled Community Advisory Groups (CAGs) with equal numbers of AA and W members who guided the study throughout. CAG meetings were conducted at the local hospital and focus groups were conducted at a local University conference room. During this phase, cultural values and preferences were determined through separate ethnic-based focus groups comprised of family members who cared for a loved one who had died within the last year. Thematic analysis focused on cultural values and preferences [47].

During phase II of the pilot, which took place at Beaufort Memorial Hospital (March 2014 to February 2016), CAG members recommended culturally appropriate programmatic implications for their ethnic group for each theme [47], and a PC physician described how these would be incorporated into the National Consensus Project (NCP) Guidelines [35] standard PC consult.

**Fig. 1:**
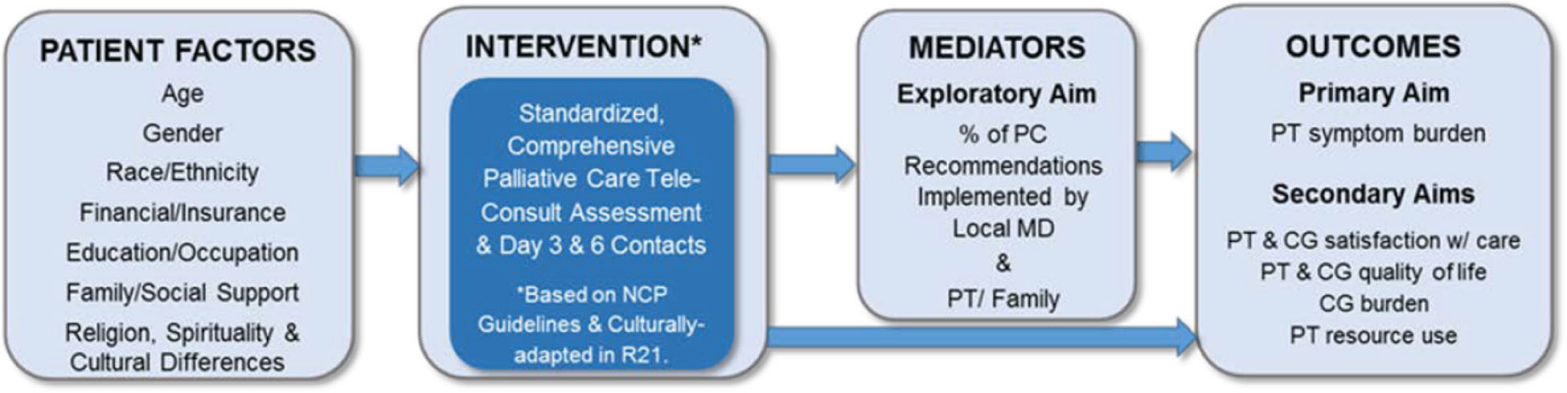
Culturally based tele-consult study model, aims, and outcomes

During phase III of the pilot, which also took place at the Beaufort Memorial Hospital, (May 2016–October 2016), we implemented the culturally based PC consultation via a secure videoconference platform for Beaufort inpatients who were referred by a hospitalist. Eligible patients were over 65 with a life-limiting illness and had a participating family care partner. Based on the feasibility of administering the intervention and family satisfaction with the intervention [47], we designed the current we developed the current study design and conceptual model (Fig. 1).

The purpose of this RCT is to compare a culturally based tele-consult program (that was developed by and for the rural southern AA and W population) to usual hospital care to determine the impact on symptom burden, patient and care partner QOL, care partner burden, and resource use post-discharge in hospitalized AA and White older adults with a life-limiting illness.

## Methods/Design

### Study overview

**Fig. 2:**
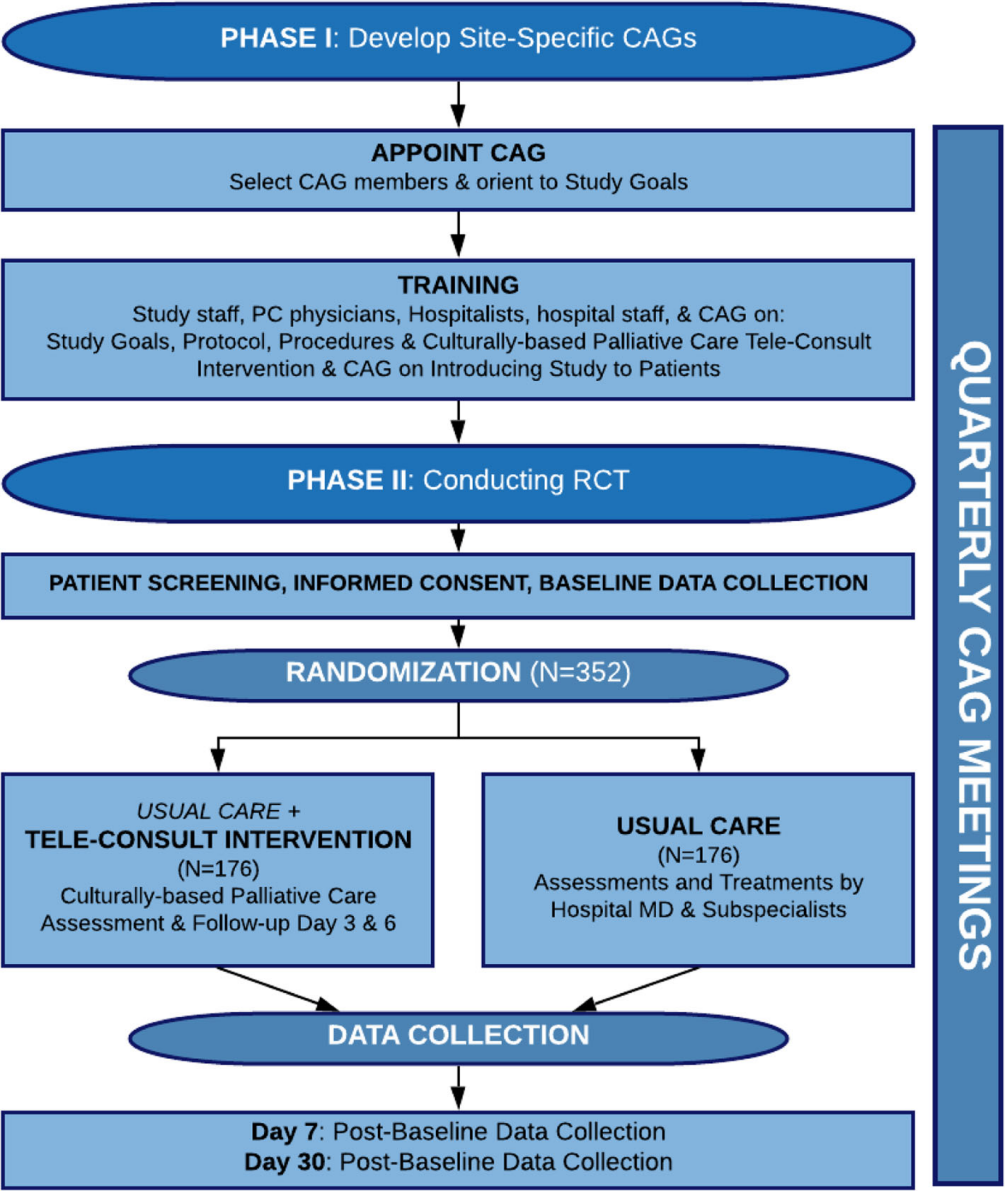
Study design

This three-site RCT will test the efficacy of a community-developed, culturally based PC tele-consult program for 352 hospitalized AA and W older adults with life-limiting illness in the Deep South. This study includes two phases, as illustrated in Fig. 2. The first phase consists of assembling CAG members to provide advice and guidance on local customs and recommendations to inform the community about the study, and how to address patients in a culturally respectful way. Additionally, during this phase, study PC physicians will be trained in their culturally appropriate communication strategies by the original CAG members who developed the original tele-consult program. Study coordinators at each of the three sites will be trained in project roles and responsibilities. In phase II, we will conduct the RCT in three rural hospitals in AL, MS, and SC to test the effectiveness of this culturally based PC program.

### Design

As depicted in Fig. 2, Community Tele-pal is a 5-year, two-phase, two-arm multi-site RCT. Phase I will engage local CAGs to help train PC physicians in the culturally based consultation approach, and in phase II, we will conduct the RCT to test the efficacy of this culturally based PC program. This study was reviewed and approved by the University of Alabama at Birmingham (IRB-300002420), Russell Medical Center (IRB-201900001), and Northwestern University (IRB-STU00209852) Institutional Review Boards.

### Setting

Phase I and phase II will be conducted at three rural hospitals in Alabama (Russell Medical Center), Mississippi (Highland Community Hospital), and South Carolina (Aiken Regional Medical Center). These rural hospitals were selected as the study sites because they had no PC services and represented typical rural hospitals in size and admission of underserved and minority populations. See Table 1 for additional information on the three participating hospitals.

**Table 1:** Three rural hospitals selected for the culturally based tele-consult intervention

Site and hospital	Description
Alabama (AL) Russell Medical Center	Located in Alexander City, AL, 80 bed, not-for-profit, acute care facility serving the needs of east central Alabama. Approximately, 24,827 patients visited the hospital’s emergency room. There are 67 physicians affiliated with the hospital, and the hospital had a total of 3245 admissions. Its physicians performed 835 inpatient and 3264 outpatient surgeries. Russell Medical Center also supports community health education services through health screenings, support groups, childbirth classes, self-help programs, and athletic trainers to multiple sports teams in its service area.
Mississippi (MS) Highland Community Hospital	Located in Picayune MS, a 60-bed not-for-profit hospital and affiliated clinics provide access to a broad range of quality services, supporting the community and enhancing the level of care for the residents of Picayune and the surrounding areas. There are 100 physicians affiliated with the Highland Community Hospital. Its community outreach includes classes and events in different themes including community education, continuing education, screenings, and support groups.
South Carolina Aiken Regional Medical Center	Located in Aiken, SC, a 245-bed acute care facility offering a comprehensive range of specialties and services. The medical staff includes over 900 skilled healthcare/support professionals, a medical staff of more than 120 multi-specialty physicians, and a team of 230 volunteers. The center provides nearly 50 specialty services through its acute care facility, behavioral healthcare hospital, and the Cancer Care Institutes of Carolina. Services are provided to residents of Aiken and its surrounding communities. Community outreach in Aiken Regional Medical Center includes classes, seminars, support groups, and information about new services.

### Phase I: developing site specific CAGs and training PC physicians and site coordinators in culturally based tele-consultation intervention and study protocol

In phase I (study year 1), the remote PC physicians will convene in Beaufort, South Carolina, to receive a 2-day training in the culturally based tele-consult and follow-up protocol by members of the original Beaufort, South Carolina CAG members. This training will include all aspects of communication strategies with particular attention to “cultural competency training” and use various feedback methods. The PC physicians will also receive specific training on the consultation template, tele-consult etiquette, use of equipment, and general study procedures.

Each site will have two part-time study coordinators: (a) blinded study coordinator (BSC) and (b) coordinating study coordinator (CSC). The study coordinators will be trained by the study principal investigators (RE, MB) in a separate 2-day face-to-face session. This training will allow coordinators to experience some team building time and will cover study design, procedures, and cultural recommendations developed by the original Beaufort, South Carolina CAG members. An evaluation will be provided at the conclusion of the training sessions to allow for feedback.

Once training for both the PC physicians and the study coordinators is complete, the study coordinators will identify and send an invitation letter to potential local CAG members in their area.

#### Community advisory group (CAG) development and training

Members of each CAG (eight total, equal AA and W) will be identified as respected members of the community who have recently experienced the loss of a loved one and understand the issues of serious illness and end-of-life care. Before study participation, each CAG member will receive hospital-specific training (similar to volunteers per hospital policy). The CAG will play three key roles:

1. Inform the study team about community culture. To accomplish this, each CAG will meet with and be trained by the original CAG members who helped develop the original culturally based PC consultation protocol.
2. Participate in quarterly face-to-face meetings with the study team to receive training, hear progress reports, and to provide input and advice with challenges or problems.
3. Introduce patients/care partners to the study by serving as the first contact for potential study participants. To accomplish this, the CAG member will accompany the study coordinator and will be the first to meet with eligible participants and their care partners to explain the community-based nature of the study.

Local CAG members will participate in an all-site CAG meeting in which CAG members will introduce themselves and receive a brief overview of the study. A second meeting will include all-site CAG members to be trained by the Beaufort, South Carolina CAG members on how the protocol was developed, what to expect, and how to introduce the study to the patient/care partner. CAG members will receive extensive training from the study PI who developed the prior study. A video highlighting the training the PC physicians received by the original CAG members about the development of the culturally based study will be shown to the CAG members so that they are able to convey the community’s role in the development of the protocol. Lastly, CAG members will devise a general flow of conversation script that will facilitate their ability to introduce the study, and will share a simplified diagram of the study that was co-created by all the CAGs, including the original CAG.

### Phase II: conducting the RCT

The aims and hypotheses of phase II are:

1. (Primary aim) To determine the impact of a culturally based PC tele-consult program on patient symptom burden in hospitalized AA and W older adults with a life-limiting illness. H1: Compared to the usual care group, patients in the intervention group will experience lower symptom burden on day 7 post-consultation.
2. (Secondary) To determine the impact of a culturally based PC tele-consult program on patient and care partner QOL, care satisfaction, and care partner burden at day 7 post-consultation, and lower resource use (hospital readmission, emergency visits) 30-days post-discharge. H2: Patients and care partners in the intervention group will experience higher patient and care partner QOL, care satisfaction, lower care partner burden at day 7 post-consultation, and lower resource use (e.g., hospital admission, emergency visits) at 30 days after discharge.
3. (Exploratory aim) Explore mediators and moderators of patient symptom and care partner burden outcomes.

#### Participants

All newly admitted non-trauma, non-elective surgery patients who are ≥65 years will be screened for eligibility. Patients are eligible if they are as follows: (1) AA or W; (2) ≥65 years of age; (3) have a condition that fits into one of three illness paradigms (e.g., cancer, chronic progressive, and fragility); (4) has a care partner who has been involved in their care; (5) able to complete baseline interviews, and (6) the clinician answers “no” to question: “would you be surprised if this person died in the next 12 months?” Patients are excluded if they are currently receiving hospice care, or no family member/care partner is willing to participate in the study. Based on the high volume of patients admitted weekly to each of the hospitals that meet eligibility, we anticipate this strategy will yield many more patients relative to recruitment goals. This conclusion was based on pre-proposal screening of admission trends.

Care partners are eligible if they are: (1) ≥18 years of age, (2) English speaking, and (3) able to complete baseline interviews.

#### Recruitment and consent

**Fig. 3:**
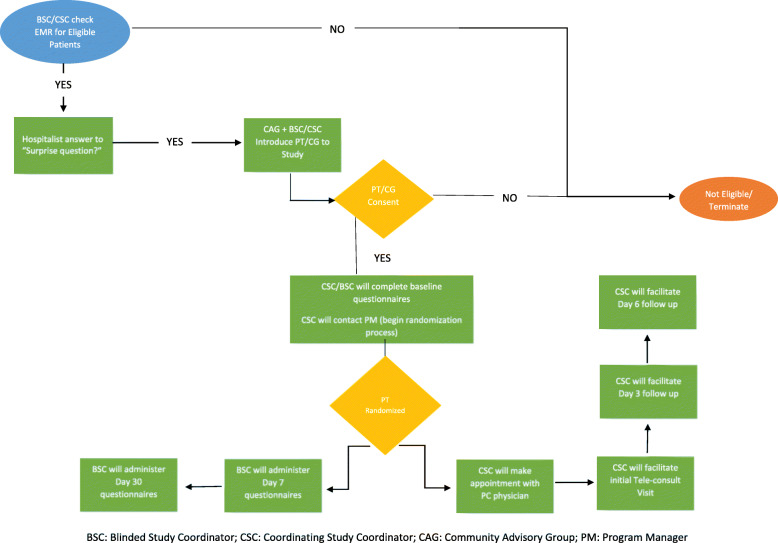
Study coordinator flow chart

Both study coordinators will screen admissions each weekday (see Fig. 3). Once a potential patient has been selected, the coordinator will ask the hospitalist at their site the “surprise question.” If the answer is no, the coordinator will contact the CAG member “on-call” and ask that they come into the hospital to introduce the study to the patient. The CAG member will emphasize their role in developing the study and how this study was designed to help their community. A CAG member, accompanied by the study coordinator, will be the first to meet and greet the eligible patients, connecting as community member to community member, and sharing the community-developed aspect of the study.

If the patient indicates that he/she would like to participate, the study coordinator will provide an overview of the study, including a description of PC, the tele-consult process, questionnaires, and randomization. If the patient expresses an interest, the coordinator will consent the patient/care partner and complete baseline questionnaires.

#### Randomization and blinding

**Fig. 4:**
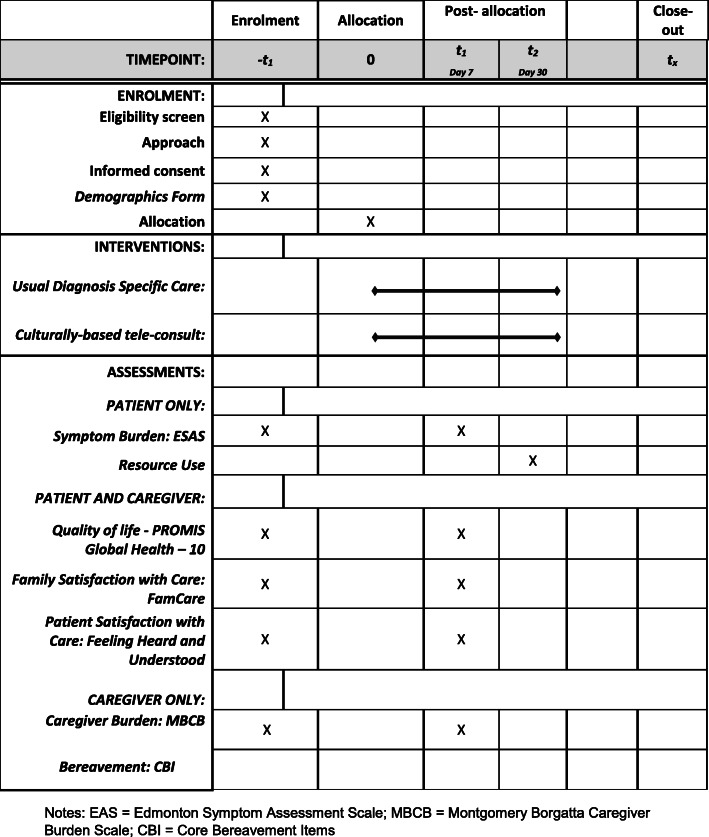
Evaluation of 2013 SPIRIT-recommended content in the community-developed, culturally based palliative care tele-consult program RCT. Notes: EAS = Edmonton Symptom Assessment Scale; MBCB = Montgomery Borgatta Caregiver Burden Scale; CBI = Core Bereavement Items

Following consent and completion of baseline questionnaires, the CSC will notify the program manager that the patient is ready to be randomized. Hereafter, the BSC will not be aware of study group assignment and will only contact enrolled patients and care partners for follow-up data collection as detailed in Fig. 3. Following patient randomization, the CSC will coordinate the timing of the first tele-consultation with the patient, family, and remote PC physician. The Standard Protocol Items: Recommendation for Intervention Trials (SPIRIT) checklist is included as an additional file. The SPIRIT diagram (Fig. 4) depicts time points for enrollment and allocation activities, intervention, and data collection.

The randomization scheme will be executed via REDCap (Research Electronic Data Capture), a clinical trial management software program. Participants will be randomly assigned to group (1:1) using a computer-generated program. The randomization scheme will be stratified by site (SC, AL, MS) and race (W, AA). As the Central Coordinating Site, UAB will manage the randomization process. The randomization will generate an email alert to the UAB program manager. The program manager will notify the local CSC to communicate the assignment to the participant and initiate the study protocol. Participants will be instructed not to discuss their assignment with the BSC who will be collecting the outcome assessments. Thus, the BSC and the data analyst/senior biostatistician will be blinded in this study, whereas the participant, the CSC, and the program manager will be aware of who has been randomized.

### The Community Tele-pal intervention

Patients randomized to the intervention group will receive the culturally based PC tele-consultation intervention using a secure video-consultation platform, in addition to the care the doctors and nurses in this hospital provide. The intervention will consist of three contacts (Table 2).

**Table 2:** Community-developed, culturally-based inpatient tele-consult intervention

Contact #	When	With whom	Method	Purpose
**#1 Palliative care tele-consult**	As soon after randomization as possible	Patient (and family) referring hospitalist and remote palliative clinician Local study coordinator Remote IDT Members	Secure tele-consult Remote IDT review eHR document	1. Conduct community-developed, culturally based palliative assessment. 2. IDT review (as needed) 3. Develop recommendations and palliative care plan
**#2 Follow-up day 3**	Within 3 days post consult	Patient and (family) Local study coordinator Remote and local clinicians	eHR document Telephone	1. Provide recommendations and care plan 2. Respond to patient, family and remote team questions 3. Identify community care team and initiate referrals as needed (primary clinician/hospice provider if appropriate).
**#3 Follow-up day 6**	Within 6 days post consult	Patient and family Study coordinator/ Remote palliative care clinician and community care team members	Telephone	1. Reassess patient and care plan 2. Assess adequacy of discharge plan or home experience 3. Refer to community resources

*eHR* electronic health record, *IDT* interdisciplinary team

#### Tele-consultation contact #1 (initial consult)

The CSC will work closely with the patient/family, local team, and remote PC physician to determine a time suitable (within 48 h on weekdays) to perform the tele-consult. The CSC and the hospitalist will be in the room with the patient and family. The CSC will set up the secure videoconferencing equipment next to the patient’s bed, introduce the patient to the remote palliative physician, and the tele-consult will proceed. Approximately 10% of tele-consultations will be recorded using a secure digital recorder for fidelity monitoring (see below). Most consults will take approximately 60 min. To ensure a comfortable conversation, we have instituted several steps to minimize interruptions including checking with floor nurse, putting up signs on the door, and tasking the CSC with running interference in case of interruptions.

Within 24 h of conducting the tele-consult, the remote palliative physician will document the consultation assessment and recommendations within the patient’s electronic medical record (eMR). They will seek interdisciplinary team input as appropriate to develop the plan and make recommendations. Recommendations may include immediate patient care (e.g., symptom relief), continuing care related to the hospital stay and discharge, and referrals to other support resources such as a hospital social worker, pastor, or hospice care. The written consult will be documented using a standardized PC consult documentation template (Table 3). Documentation will occur as part of the patients’ inpatient medical record. During the consultation, PC physicians will utilize protocol prompts (Table 4) that correspond to the eight NCP PC domains [35] and the culturally based recommendations that the original Beaufort, South Carolina CAG members helped to develop. As needed, verbal contact will also occur between the PC physician and the hospitalist staff.

**Table 3:** Palliative care consult documentation template (entered into eHR)

**Hospital Name [AUTOPOPULATE, if possible]**	
**Date of consult: [AUTOPOPULATE, if possible]**	
**Patient Name/DOB/Gender/Race/MR# [AUTOPOPULATE, if possible]**	
**Referral Source/Provider: [AUTOPOPULATE, if possible – if considered an “order”]**	
**Primary problem/focus of consult: [If possible, havedrop-down listwith these options:** **• Symptom Management** **• Support/Coping** **• Goals of Care/Advance Care Planning** **• Interdisciplinary referral(s)** **• Local Resources / Community Care Medical / Other Support Communication** **• Hospice / Home Services** **• Other]**	
**Secondary problem/focus of consult: [If possible, havedrop-down listwith these options:** **• Symptom Management** **• Support/Coping** **• Goals of Care/Advance Care Planning** **• Interdisciplinary referral(s)** **• Local Resources / Community Care Medical / Other Support Communication** **• Hospice / Home Services** **• Other]**	
**History of Present Illness:**	
**Past Medical/Surgical History: [AUTOPOPULATE, if possible]**	
**Medications Review and Allergies: [AUTOPOPULATE, if possible]**	
**Physical ROS/Cognition/Functional status:**	
**Social History/Assessment:**	
**Support Systems/Family concerns:**	
**Spirituality/Beliefs:**	
**Physical Exam-Limited: [AUTOPOPULATE Vital signs, Weight, Inputs/Outputs (including bowel movements) if possible]**	
**Lab/Diagnostic studies/Records Review Highlights: [DO NOT AUTOPOPULATE]**	
**Palliative Performance Scale: [if possible, have table below displayedANDdrop-down with choices:** **• 100% = Ambulation: Full; Activity & Evidence of Disease: Normal activity & work, No evidence of disease; Self-Care: Full; Intake: Normal; Conscious Level: Full** **• 90% = Ambulation: Full; Activity & Evidence of Disease: Normal activity & work, Some evidence of disease; Self-Care: Full; Intake: Normal; Conscious Level: Full** **• 80% = Ambulation: Full; Activity & Evidence of Disease: Normal activity with Effort, Some evidence of disease; Self-Care: Full; Intake: Normal or reduced; Conscious Level: Full** **• 70% = Ambulation: Reduced; Activity & Evidence of Disease: Unable Normal Job/Work, Significant disease; Self-Care: Full; Intake: Normal or reduced; Conscious Level: Full** **• 60% = Ambulation: Reduced; Activity & Evidence of Disease: Unable hobby/house work, Significant disease; Self-Care: Occasional assistance necessary; Intake: Normal or reduced; Conscious Level: Full or Confusion** **• 50% = Ambulation: Mainly Sit/Lie; Activity & Evidence of Disease: Unable to do any work, Extensive disease; Self-Care: Considerable assistance required; Intake: Normal or reduced; Conscious Level: Full or Confusion** **• 40% = Ambulation: Mainly in Bed; Activity & Evidence of Disease: Unable to do most activity, Extensive disease; Self-Care: Mainly assistance; Intake: Normal or reduced; Conscious Level: Full or Drowsy +/− Confusion** **• 30% = Ambulation: Totally Bed Bound; Activity & Evidence of Disease: Unable to do any activity, Extensive disease; Self-Care: Total Care; Intake: Normal or reduced; Conscious Level: Full or Drowsy +/− Confusion** **• 20% = Ambulation: Totally Bed Bound; Activity & Evidence of Disease: Unable to do any activity, Extensive disease; Self-Care: Total Care; Intake: Minimal to sips; Conscious Level: Full or Drowsy +/− Confusion** **• 10% = Ambulation: Totally Bed Bound; Activity & Evidence of Disease: Unable to do any activity, Extensive disease; Self-Care: Total Care; Intake: Mouth care only; Conscious Level: Drowsy or Coma +/− Confusion** **• 0% = Death**	
**Goals of Care / Advance Care Planning Assessment:**	
**Global Assessment Statement:**	
**Recommendations/Plan:** **A. Symptom Management** **B. Support / Coping** **C. Goals of Care / Advance Care Planning (inc. AD, Proxy, DNR, POLST, etc.)** **D. Interdisciplinary Referrals (PT, OT, Spiritual, Counselor, Social Work, etc.)** **E. Local Resources / Community Care Medical / Other Support / Communication** **F. Hospice / Home Services** **G. Other**	
**Transition/Discharge Plans (if known):**	

*DOB* date of birth, *MR* medical record, *ROS* review of symptoms

**Table 4:** Protocol prompts

African American	White
**Distrust is ever present.** Recognize and respect. Work to establish trust.	
**White coat, tele-health**: Wear white coat	
**Tele-health**: Acknowledge not same as face to face	
**Address patient/family**	
By last name or title ONLY	
1. Introduce self, then invite patient/family to introduce self, hospital staff and CRC last	
**Rapport building**	
Get to know patient, begin establishing rapport	
Take additional time to get to know family	
Learn specifics about family and talk about it	
Discuss something local	
**Family care/social history**	
Recognize that family will be there for patient and care for them at home. Start with that assumption.	
**Illness understanding/prognosis discussion**	
1. Ask patient/family if want to know prognosis;	1. Sensitively determine if patient/family want to know about prognosis.
2. Never be blunt.	2. Honor their decision (i.e., if do not want to know, do not discuss and vice versa).
3. Never tell patient they are dying.	3. Be a part of their journey.
4. If family asks prognosis, never give date or time, only range.	
5. Explain what’s happening in the body very simply (no medical terms).	
6. Offer opportunity for patient and family to ask questions. If family does not understand, explain in different way	
7. If patient/family is religious, physician can say, “I can see that you are a spiritual person, we are doing the best that we can and it’s in God’s hands.”	
8. *Alway*s say: it’s in God’s hands/God decides. If physician not comfortable saying, “God”, say, “it’s in the hands of a higher power.”	
9. If physician is comfortable, ask if you can pray with the patient/family.	
**Medications/symptoms**	
1. Explain why pain meds needed, especially morphine dosing	
2. If concern about lack of consciousness raised, explain balance between pain free and being asleep/unconscious	
3. If concern about morphine dose change, explain flexible dosing	
4. If concern about addiction is raised, explain addiction not problem	
5. If fear of overdosing is raised (with potential to enhance death), address concern and ease fear.	
6. Explain simply; no medical language.	
**Goals of care, treatment preferences and ACP**	
May be confused between: AD, DNR, Power of Atty.	
Recognize: Care instructions given verbally to family	1. Ask if they have any documents of wishes.
1. If patient cannot communicate: Ask if shared instructions w family (and who)	2. Ask if have been asked to complete documents. Clarify if questions
2. Ask family what care patient wanted	3. If has document, ask: What does it specify? Have they changed? Has hospital followed them?
	4. If no AD, ask if know what care pt. wanted
	5. If not AD, ask if want to complete one.
**Role of religion and church**	
Recognize importance of pastors, especially in discussing prognosis. Invite pastor to next meeting if discussing prognosis.	
Recognize importance of religion, source of comfort, knowledge, a guide for all things	Recognize church members are a source of support. If support needed, ask if a church member can assist; ask name of church member and discuss how they can provide support.
**Financial vulnerability**	
Recognize may experience substantial financial difficulties, with harsh/challenging realities	
**Death and dying**	
Recognize death is not discussed in church. Approach possibility of death with caution.	
**Hospice**	
1. Never say ‘hospice’ and do not raise it UNLESS the patient/caregiver raises it or expresses concern about burden of care OR asks about hospice.	1. Assess how patient and family feel about hospice but do not use the word, “hospice.” Use “home health.”
2. Ask which family members are helping to take care of patient (and how). If it is the kind of care home hospice provides, explain that this is the type of care that home health provides.	2. Whatever their response, acknowledge and respect their feelings/attitudes.
3. Ask if there are any specific concerns (e.g. cleaning a port, bathing a patient with an open wound) about the family providing care, and discuss until all concerns are alleviated.	3. If open to it, talk about this is a helpful way to take care of the family at home.
4. Emphasize that home health NOT there to take over; the family is in charge.	4. Make sure to emphasize that this is an offer of help and assistance.
5. If open to it, talk about how this is a helpful way to take care of the family at home.	
6. Ask if have any concerns about this kind of home help. Address concerns	
7. Acknowledge and respect their feelings/attitudes.	
8. If patient/family wants home health/hospice, ask if want you to make referral.	
9. Stress that all decisions are up to the patient/family. You’re there only to help.	
**Nursing homes**	
1. If patient in nursing home, or family/patient raises issue, discuss nursing home referral. Do not raise if they do not	1. If patient is in nursing home, help family deal with guilt
2. If loved one is going to nursing home, provide support to family.	

*AD* advance directive, *DNR* do not resuscitate

#### Tele-consultation contact #2 (day 3 follow-up; approximately 48–72 h after first contact)

The CSC will arrange a follow-up videoconference between the remote PC physician and patient if they are in the hospital or via phone if the patient has been discharged. The purpose of this second contact is to determine how the patient is progressing, if the plan was implemented, and if hospital staff/patient has encountered any challenges or questions and to determine who will be assuming the patient’s care in the community. Documentation of the contact will occur in the eMR if the patient is still hospitalized. Otherwise, it will be completed in the secure RedCap study database and will be provided to appropriate community care providers.

#### Tele-consultation contact #3 (day 6 follow-up; via videoconference if patient is in hospital, via phone if discharged)

The CSC will determine if the patient is experiencing new or ongoing challenges and confirm ongoing availability of community or hospice care as appropriate.

### Usual care

Patients randomized to the usual care group will receive inpatient care appropriate to their illness and consistent with their care prior to enrolling in study. This includes assessment and treatment by the admitting physician (hospitalist), along with any subspecialists that are consulted. The formulation and sharing of prognostic information and the engagement of patients and families in establishing goals of care will be according to the standards of the admitting physician and subspecialists.

### Outcome measures

At each data collection point, the following measures will be administered: patient symptom burden (primary aim) and patient-care partner-reported responses regarding QOL, satisfaction with care, and care partner burden (secondary aims). Additionally, at 30 days post-baseline, resource use (hospital admission, emergency department visits, and hospice days) will be tracked via electronic health record review and patient/family report (secondary aims). If a patient participant dies within the day 30 window, usual care and the intervention group care partners will be asked to complete a bereavement questionnaire by phone.

### Data collection

Following baseline data collected at the time of consent, BSCs will administer questionnaires to the patient and their designated care partner, in person or by telephone (if patients are discharged during the study period) on day 7 (T2) and day 30 after hospital discharge (T3). Table 5 outlines the three-time points and outcome measures for this study. The CSC and BSC will assist the patient (and separately the family member) to complete the baseline (T1) questionnaires using a tablet that connects directly to the REDCap database or using paper questionnaires if the participants prefer. Coordinators have undergone extensive training in administering and entering questionnaires in the REDCap database following participant completion. Additional measures to promote data quality and completeness are embedded in the REDCap database. For example, the study coordinators are not permitted to skip questions or forms that are vital to eligibility and outcome measures.

**Table 5:** Outcome measures

	Domain	Specific measurement	Description of measure	Schedule
**Primary aim**				
**Patient**	Symptom burden	Edmonton Symptom Assessment Scale (ESAS)	Symptom intensity using visual analog *(9 items)*	Baseline, day 7, day 30
**Secondary aims**				
**Patient**	QOL	PROMIS Global Health-10	Evaluates physical, social, and emotional health in healthy and chronically ill adults *(10 items)*	Baseline, day 7, day 30
	Satisfaction	Feeling Heard and Understood	Self-report quality measures for palliative care settings on Likert scale *(1 item)*	Baseline, day 7, day 30
	Resource use		Patient resource use (hospital readmissions, # of hospital days, # of ICU days etc.)	Day 30
**Caregiver**	QOL	PROMIS Global Health-10	Evaluates physical, social, and emotional health in healthy and chronically ill adults *(10 items)*	Baseline, day 7, day 30
	Satisfaction	FamCare	Family satisfaction with availability of care, physical, and psychosocial care, information giving *(20 items)*	Baseline, day 7, day 30
	Caregiver burden	Montgomery Borgatta Caregiver Burden Scale (MBCB)	Subscales objective, subjective, demand burden *(14 items)*	Baseline, day 7, day 30
**Exploratory aim**				
**Patient**	Symptom burden	Edmonton Symptom Assessment Scale (ESAS)	Symptom intensity using visual analog *(9 items)*	Day 7
**Caregiver**	Caregiver burden	Montgomery Borgatta Caregiver Burden Scale (MBCB)	Subscales objective, subjective, demand burden *(14 items)*	Day 7
**Caregiver**	Bereavement	Caregiver Evaluation of Quality of End-of-Life (CEQUEL)	*13 items*	2–3 months
	Bereavement	Core Bereavement Items (CBI)	Subscales/Item *(7 items)*	2–3 months

### Statistical analysis plan

Demographic variables (e.g., age, sex, race, disease) will be summarized and compared across treatment groups using *t*-tests and nonparametric Wilcoxon signed rank tests for continuous measures (age), binomial tests (sex, race), and chi-square statistics for categorical variables (e.g., marital status, education). All demographics and stratification variables (site and race) found to differ across treatment groups will be included in regression models; however, race will be included in the final models. Tabulations of binary and categorical variables will be presented for all measures and broken down by treatment group. Age will be summarized by mean and standard deviation for all participants and broken down by treatment group.

For patients, we will conduct longitudinal (intention to treat) analysis of the primary study outcome, symptom burden (measured by ESAS). Symptom burden will be assessed for change in longitudinal regression models from baseline to day 7. Patients are being randomized by site to account for community-level factors. There is only one palliative care doctor for each site, so we are unable to account for this. Regarding hospitalists at each site, we will document which hospitalist is caring for the patient. We will examine if hospitalist physician affects outcomes.

Scores for secondary outcome measures (patient satisfaction with care, care partner satisfaction with care, patient QoL, care partner QoL) will be analyzed using the same strategy described for the primary outcome. Resource use (e.g., hospital admissions and the number of emergency room visits) will be assessed from the day of patient’s hospital discharge until 30 days later. Patterns of missing data will be assessed and models will be adjusted for any factors associated with missingness.

Mediation analysis will follow the approach of Kohler, Karlson, and Holm [48]. Possible binary mediators include the implementation level of PC physician recommendations by (1) the treating clinician, and (2) the patient/family. Using this approach, we will infer results that are decomposed into direct and indirect effects. If there is no evidence of mediation, only direct effects will be estimated in appropriate models.

### Sample size and power considerations

Assuming a uniform accrual of patients, we will enroll 352 patients stratified by site and race using a block randomization method into two groups (176 per treatment group). This sample size is similar to a study led by Bakitas and colleagues which enrolled 322 patients. The sample had sufficient power to detect and report meaningful results in measures such as QOL, symptom intensity, and depression scores [5]. This sample size will result in 80% power at 0.025 (to adjust for multiple comparisons in interaction models) to detect a 0.33 standardized effect.

### Community Tele-pal intervention fidelity monitoring

**Fig. 5:**
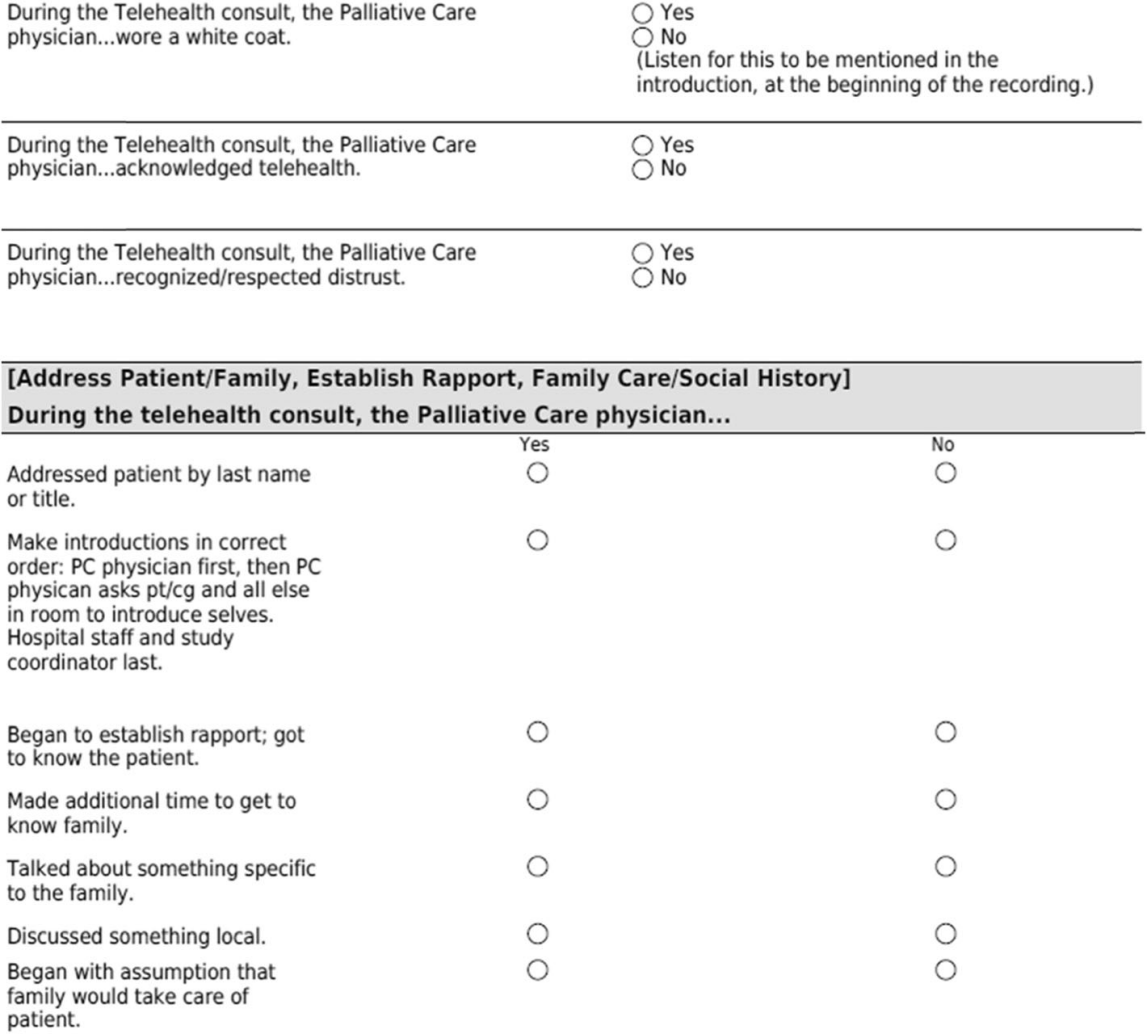
Culturally based tele-consult intervention fidelity monitoring

As previously mentioned, 10% of the tele-consult visits will be digitally recorded to assess for intervention fidelity. The CSC or the BSC will be assigned a recording of a PC physician from a different site. The CSC or the BSC will use the fidelity checklist (Fig. 5) to monitor the palliative physicians’ adherence to the study protocol by rating the digital audio recording of the tele-consult sessions and the consult documentation. Randomly selected participants will have all three of their protocol contacts reviewed. If ratings are below the “satisfactory” rating on the scales, supervision will focus on necessary techniques to improve the score to satisfactory or higher.

### Usual care monitoring

Patients randomized to usual care in each of the three participating hospitals will receive inpatient care appropriate to their illness. This includes assessment and treatment by the admitting physician, along with any consultations from subspecialists. The formulation and sharing of prognostic information and the engagement of patients and families in establishing goals of care will be according to the standards of the admitting physician and subspecialists.

We will review usual care patients’ medical records during hospitalization 30 days post-baseline and will document the patients’ use of any supportive care or medical services during that time. This will allow us to track practice, care, and resource use patterns over time to examine temporal trends or changes and to identify any potential contamination, such as an increase in supportive care services and hospice,. over the course of the study.

### Ethical aspects

Amendments reflecting study modifications were submitted for institutional review board approval at all sites and updated on the clinical trial registry. The initial study protocol (version 1.0) was approved in 2019 and underwent minor modifications (version 1.1, 1.2, 1.3), which were approved in February, June, and October respectively. The protocol went through continuing review (version 1.4) and was approved in January 2020.

Study coordinators will securely and confidentially manage all data and personal health information in accordance with Health Insurance Portability and Accountability Act (HIPAA) and IRB regulations. The only physical study documents that will contain identifiable information will be the informed consent and PC template documents. The CSC and program manager will store these documents in secure, locked locations and a password-protected secure server. Paper copies of questionnaires, fidelity checklists, data collection sheets, spreadsheets, and any other data collection instruments and tools will be kept in locked and secure physical and electronic locations by the CSC and BSC. These will then be securely transmitted to the UAB program manager. The MPIs will follow the NIH policy for data and safety monitoring. The data and safety of the study will be monitored annually by the Data Safety Monitoring Committee, which will include faculty from UAB School of Medicine. The data and safety monitoring committee will be comprised of physician and nurse experts, and an external statistician who are independent of the protocol. They will oversee the conduct of the intervention (including any adverse events or protocol deviations, data collection and analysis, and review blinded demographic and patient-reported outcomes).

Participants are free to withdraw from the study at any time. This study has minimal risk to participants. Some risks associated with participating in the study include emotional distress, loss of confidentiality, and burden versus benefits.

- Emotional distress: talking about their illness and symptoms may cause emotional distress for patients or care partners. Patient may get tired while answering the questions with the study coordinator. If a patient gets too tired, we can come back later in the day. Sometimes people feel embarrassed or uncomfortable when being asked questions so patients/care partners can refuse to answer any question. Upon participant report of emotional distress, data collection can be paused and resumed at a later date. The study coordinators will follow-up to ensure the participant feels supported and this distress will be reported to the palliative care clinician or inpatient support services if necessary.
- Loss of confidentially: There is a chance that people not associated with the study will see patient/care partner's answers to questionnaires. Therefore, any identifying information will be removed from study documents. Data will be kept in locked files in the study research offices at UAB with study ID numbers. All data will be housed in a secure, password-protected database at UAB.
- Burden versus benefits: There is a risk related to being placed into a group by chance (i.e., randomization). Patients in the usual care group may not have the same benefits as patients in the intervention group during their inpatient stay. However, both groups may receive outpatient palliative services from a local home hospice organization if they are available and deemed appropriate by the clinicians and patient/family. Because this trial is being conducted in a facility that lacks palliative care services, there are no inpatient palliative care services available to usual care patients. However, to the extent they wish to receive palliative care from an external hospice agency after hospital discharge, that will be determined by the hospitalist and the patients/family. There are also no scheduled intervention palliative care contacts after contact #3; however, as for the usual care group, palliative care services from home hospice agencies may be instituted.

Information obtained about the patient/care partner will be kept confidential to the extent allowed by law. However, research information that identifies the patient/care partner may be shared with people or organizations (i.e., UAB IRB, NINR, or the Office for Human Research Protections) for quality assurance or data analysis, or with those responsible for ensuring compliance with laws and regulations related to research. The datasets that will be used and analyzed during the current study will be available from the corresponding author on reasonable request.

There are no future studies planned that would require the participant to be reconsented. Upon completion of the study, findings will be submitted to the appropriate national organizations, conferences, and seminars. Additionally, manuscripts will be submitted to the appropriately selected journal. All study personnel will be included as appropriate to their level of effort in producing manuscripts. First and senior author positions will be alternated among multi-PIs and co-investigators commensurate with their effort. We will not be using any professional writers to draft manuscripts. Plans for public access to protocol, participant data, and code will be allowed as required by the granting agency.

## Discussion

Rural-dwelling, seriously ill older adults have poor access to PC in the Southeastern United States [20]. This translates to poor-quality end of life care and high cost due to multiple institutional and culturally based factors [49–53]. Conducting research in small rural hospitals can be difficult. PC is considered foreign, and there can be misconceptions and lack of trust from “outsiders,” especially when the physician will not physically be present. Potential barriers to success include participant recruitment, usual care contamination, and participant attrition.

To increase participant enrollment and recruitment, the study investigators and PC physicians have worked to build a relationship with the chief hospitalists at each site. We have also done extensive training with study coordinators to help refine recruitment procedures to minimize selection bias (e.g., all admissions will be screened for eligibility criteria rather than relying on physician referral). Extensive training was also provided to CAG members, who will be the first to meet with the patients and care partners. This was recommended by the AA CAG members who developed this culturally based program as a way to reduce potential distrust of study participation by AA patients, by explaining that this program was developed by community members like them. The W CAG members also adopted this method.

Usual care contamination is possible because treating clinicians have patients in both groups. However, research suggests that treating clinician behavior change is unlikely merely based on observing suggested practices [54]. Also, because the chief hospitalists have a role in the study, they may be more likely to adhere to the study protocol. However, we will be monitoring the usual care participants for temporal trends related to changes in care practices such as more frequent referral to hospice or supportive care services once the study is underway.

To promote participant retention and reduce potential loss and to enhance complete data collection efforts, participants will receive a monetary incentive at each data collection time point. We hope this will encourage continued involvement with data collection until 30 days post-discharge to minimize missing data and attrition rates. Furthermore, the study coordinators will establish relationships with patients and care partners to engender their commitment to completing data collection over 30 days.

Culturally developed PC programs have the potential to significantly reduce health disparities related to PC engagement for rural Americans. The public health consequences arising from rural living can be categorized into access and acceptability. To our knowledge, this study addresses these two urgent public health gaps: determining methods to provide effective PC to rural communities, and doing this in a culturally based manner. We expect that this RCT will address critical knowledge gaps in finding effective ways of (1) providing PC to rural areas and (2) investigating the efficacy of a culturally based PC consult program developed by and for the populations (AA and W rural southern patients) it will serve. If found to be effective, the model for developing culturally based PC programs can be used to create and test programs that are culturally based for other rural areas and other population groups.

## Trial status

Community Tele-pal is an ongoing PC RCT. Recruitment will begin on July 15, 2020. At the time of submission of this manuscript, key personnel completed phase I of the study and the RCT were about to begin at one of the participating sites. We will actively recruit patients and care partners until May 29, 2022. At the time of this submission, the protocol is on version 1.4.

## Supplementary information

**Additional file 1.** Sample Consent Form for Patient and Caregivers.

**Additional file 2.** SPIRIT 2013 Checklist: Recommended items to address in a clinical trial protocol and related documents.

## Data Availability

Not applicable, no datasets are included in this study protocol.

## Abbreviations

AA: African American
ARMC: Aiken Regional Medical Center
CBI: Core Bereavement Items
CEQUEL: Caregiver Evaluation of Quality of End-of-Life
ESAS: Edmonton Symptom Assessment Scale
FamCare: Family satisfaction with care
HCH: Highland Community Hospital
MCBC: Montgomery Borgatta Caregiver Burden Scale
NA: Not applicable
NCP: National Consensus Project
NIH: National Institutes of Health
NINR: National Institute of Nursing Research
PC: Palliative care
PROMIS: Patient-Reported Outcome Measurement Information System
QOL: Quality of Life
RCT: Randomized controlled trial
REDCap: Research Electronic Data Capture
RMC: Russell Medical Center
SPIRIT: Standard Protocol Items: Recommendation for Intervention Trials
UAB: University of Alabama at Birmingham
W: White
