# Supplementary material for: Community Tele-pal: A community-developed, culturally based palliative care tele-consult randomized controlled trial for African American and White Rural southern elders with a life-limiting illness
Source: Trials. 2020 Jul 23;21:672. doi: 10.1186/s13063-020-04567-w (PMC7376880; doi:10.1186/s13063-020-04567-w)
Supplement: Supplementary file 1 — Additional file 1. Sample Consent Form for Patient and Caregivers. [file 13063_2020_4567_MOESM1_ESM.docx]

Additional file 1: Sample Consent Form for Patient and Caregivers

**PATIENT CONSENT FORM**

**Title of Research:** A Community-Developed, Culturally-Based Palliative Care Tele-Consult Program for African American and White Rural Southern Elders with a Life Limiting Illness

**UAB IRB Protocol #:** IRB-300002420

**Principal Investigator:** Ronit Elk, Ph.D., and Marie Bakitas, DNSc

**Sponsor:** National Institute of Nursing Research/NIH/DHHS

| **General Information** | You are being asked to take part in a research study. This research study is voluntary, meaning you do not have to take part in it. The procedures, risks, and benefits are fully described further in the consent form. |
| --- | --- |
| **Purpose** | The purpose of the study is to determine if palliative care delivered by a teleconsult visit is better than usual hospital care, without a palliative care teleconsult visit, in improving quality of life and management of symptoms, and reducing caregiver burden, for patients aged 65 and over in the rural South who are admitted to the hospital. There are no palliative care doctors in your area, but the teleconsult visit allows us to bring this special type of care to patients who would otherwise not be able to get it. ***Palliative care*** is a medical specialty that is concerned with improving patient’s quality of life and symptoms. Palliative care doctors work with the patient’s other medical doctors and health professionals to provide patients and families with an extra layer of support while they are dealing with a chronic illness. A ***teleconsult*** visit provides a typical evaluation by a specialist doctor; however, instead of the doctor visiting you in your hospital room in-person, we will use a computer screen (like a tablet) to allow you and whomever else is in your room and the doctor to see each other through the screen (like Skype or FaceTime, except our video has special security to protect your privacy). |
| **Duration & Visits** | You will be in this study for 30 days. Depending on which group you are assigned to, participants in the usual care group will have the medical care and supportive services that would be provided to any hospital patient. Participants in the intervention group will receive the medical care and supportive services that would be provided to any hospital patient in addition to a teleconsult visit (duration approximately 45 minutes) with a palliative care doctor that’s been developed for patients in the rural South.  Both groups will complete questionnaires at **3** time points:  • On the day of enrollment (Day 0): 40 minutes  • Day 7: 20 minutes  • Day 30: 20 minutes |
| **Overview of Procedures** | If you are eligible for the study, you will be approached in your hospital room by a study coordinator and a local community member who will introduce you to the study while you are in the hospital. If you agree to participate in the study, a coordinator will ask that you read this document and sign at the end. The coordinator will then ask that you complete baseline questionnaires. After you complete these baseline questionnaires, you will be randomized (placed into a group by chance, like a flip of a coin) to either the usual care group or the palliative care intervention group.  The usual care group will receive medical care and supportive services that are provided to any hospital patient. The intervention group will receive the same care that is provided to any hospital patient in addition to receiving a teleconsult visit from a palliative care doctor. On Day 3 and Day 6 after the teleconsult visit if you are still in the hospital the coordinator and/or palliative care doctor will contact you in person or by phone to check on how you are doing and be sure you have the resources you need when you leave the hospital. If you are discharged from the hospital on Day 3 or Day 6 the coordinator and/or MD will contact you by telephone using the contact information you will provide before you are discharged.  On Day 7 and Day 30, a different coordinator from the one that helped with the teleconsult will follow up to collect data by asking you questions about how you’re feeling and how satisfied you were with the care you received in the hospital. These questions are the same or similar to the ones you were asked upon study enrollment. |
| **Risks** | This study has minimal risks. Some participants may feel anxious or bothered by the questionnaires. We have made these questionnaires as brief and simple as possible. If you don’t feel comfortable answering a question, you may leave it blank. Another risk is loss of confidentiality but we have protections in place to reduce the possibility of this happening. Lastly, there is risk related to being randomized. |
| **Benefits** | You may or may not directly benefit from participating in this study. However, this study may help us better understand how to care for African American and white patients, with chronic illness, who are 65 years and older and their caregivers in the rural South. Also, participants in the intervention group may benefit by talking to a palliative care doctor who has been trained by community members who participated in developing the program in how to understand, talk to, and treat African American and White people in the rural South. |
| **Alternatives** | The alternative is to not participate in this study. |

## **Purpose of the Research Study**

You are being invited to participate in a research study. Taking part in research is voluntary. You will continue to receive the care prescribed by your doctors at the hospital whether you choose to participate or not. Participation in this research program will be in addition to that care. In order to participate we will also ask you to identify a caregiver. This someone who knows you well and is involved in your medical care. This form explains what you will be asked to do if you and your caregiver decide to participate in this study. Your caregiver will be asked to sign a separate consent form that explains what we are asking them to do. This form can be read aloud if you prefer. If you decide to participate in this study, you will be asked to sign this form. We will give you a signed copy of this form to keep. Please ask questions if there is anything you do not understand.

The purpose of the study is to determine if palliative care delivered by a teleconsult visit is better than usual hospital care, without a palliative care teleconsult visit, in improving quality of life and management of symptoms, and reducing caregiver burden, for patients aged 65 and over in the rural South who are admitted to the hospital. There are no palliative care doctors in your area, but the teleconsult visit allows us to bring this special type of care to patients who would otherwise not be able to get it. Your hospital doctor will remain in charge of your care and will take the palliative care doctor’s recommendation into consideration in providing your care.

The goal of this study is to see if receiving a palliative care teleconsult, in addition to the care provided by your doctors and nurses at this hospital, improves patient symptoms. There will be 352 patients across 3 hospitals (Alabama, South Carolina, and Mississippi) who will participate in the study. Half of the participants in this study (Group A) will receive the usual care that doctors and nurses at this hospital will provide for you. The other half of the participants in the study (Group B) will receive the palliative care teleconsult that’s been developed for patients in the rural South, in addition to the usual care that the doctors and nurses in this hospital provide.

## **Study Participation & Procedures**

If you agree to join the study, you and your caregiver will go through the following steps:

1. **Consent Form**: If you and your caregiver agree to enroll in this research study, a study coordinator will explain the study and answer any questions you have. Then, you will be asked to sign this consent form and your caregiver will be asked to sign a separate consent form.
2. **Questionnaires**: The study coordinator will ask you to answer some questions about how you feel and how things are going for you. This will take about 40 minutes.
3. **Group Assignment**: After you, and your caregiver, complete the first set of questionnaires with the study coordinator, you will be assigned by chance (like the flip of a coin) to one of the two study groups (either Group A – usual medical care, or Group B – usual medical care and teleconsult visit). No one can control which group you will be assigned, not the doctor, the study coordinator, the nurse or you; it is done by a computer program. One of the study coordinators will not know which group you have been assigned to. This is called blinding. You will be asked not to tell the coordinator which group you are assigned to.

**If you are assigned to Group A – *Usual medical care*:**

- **Medical Care**: Patients in Group A receive the medical care and supportive services that would be provided to any hospital patient.
- **Questionnaires**: One study coordinator will be responsible for collecting future questionnaire on Day 7 and 30. They will not know which group you have been assigned to. This is called blinding. You will be asked not to tell the coordinator which group you are assigned to. They will collect these questionnaires on Day 7 (questionnaires will take approximately 20 minutes) and on Day 30 (questionnaires will take approximately 20 minutes).

**If you are assigned to Group B – *Usual medical care AND teleconsult visit*:**

- **Medical Care**: Patients in Group B receive the medical care and supportive services that would be provided to any hospital patient and the palliative care teleconsult visit.

If you are in this group, a study coordinator will schedule a teleconsult meeting for you with the palliative care doctor, at a time that is convenient for you and your caregiver either later today or tomorrow. For your appointment, the study coordinator will bring in a special screen (computer or tablet) and introduce you to the palliative care doctor (the study provides all of this equipment – you will not need to have a computer, tablet, or smartphone to participate). You will be able to see and hear the doctor and she or he will be able to see and hear you. The palliative care teleconsult visit will take place in your hospital room with you and your caregiver. The study coordinator will stay in the room with you to make sure the screen is working well and provide assistance. The appointment with the palliative care doctor may take up to 1 hour. At the end of the appointment the palliative care doctor will discuss their recommendations for your treatment with you and your caregiver.

**Please note some of the tele-consult visits will be audio recorded using a secure device for quality reasons.** We will notify you in advance if your visit will be one of the ones we wish to record. We will only be audio recording the teleconsult visit conversation. The recording will only be reviewed by a trained member of the study team to make sure the palliative care doctor is covering all of the important points during the visit. After all study activities and data analysis are completed, all recordings will be destroyed.

- **Palliative care report to your doctor**: Shortly after the teleconsult visit, the palliative care doctor will put a note, of his or her recommendations that he or she discussed with you and your caregiver, into your electronic medical chart at the hospital for your hospital care team to see. He or she may also call your hospital doctor to talk through the recommendations.
- **Your hospital doctor**: Your hospital doctor will read over the recommendations made by the palliative care doctor, discuss these recommendations with the palliative care doctor, and determine which recommendations are right for you.
- **Contact #2 Day 3** (approximately 72 hours after the teleconsult visit with the palliative care doctor): Depending on whether you are still in the hospital or have been discharged, a study coordinator will arrange a follow-up video call (if still in the hospital), or phone call (if discharged), between the palliative care doctor and you. The purpose of the second contact is to determine how you are feeling, if the palliative care doctor’s plan was implemented, if you have encountered any challenges or questions, and to determine who will be assuming your care in the community, outside of the hospital.
- **Contact #3 Day 6** (approximately 6 days after the teleconsult visit with the palliative care doctor; by video if you are in hospital, by phone if discharged): The purpose of the third contact is to determine if you are experiencing new or ongoing challenges and to confirm availability of community or hospice care as appropriate.
- **Questionnaires**: ***Day 7 and Day 30*** - One study coordinator (who did not help with the teleconsult) will be responsible for collecting future questionnaires on Day 7 and 30. They will not know which group you have been assigned to. This is called blinding. You will be asked not to tell the coordinator which group you are assigned to. They will collect these questionnaires on Day 7 (questionnaires will take approximately 20 minutes) and on Day 30 (questionnaires will take approximately 20 minutes).

| **TIMELINE** | | | | | |
| --- | --- | --- | --- | --- | --- |
| **When** | **Where** | **What** | **Group A** | **Group B** | **Approx.**  **Duration** |
| Today (Day 0) | In-person, in your hospital room | 1. Review and sign consent form  2. Complete first set of questionnaires | X | X | 1. Consent: 35 min  2. Questionnaires: 40 min  **Total**: up to 1 hour and 15 minutes |
| Today or tomorrow | In-person, in your hospital room | Teleconsult visit with a palliative care doctor |  | X | 45-60 minutes |
| Day 3 | **In-person**, in your hospital room, **or by phone**, if you have been discharged | Contact by a study coordinator and/or palliative care doctor to see how you are doing |  | X | 20 minutes |
| Day 6 | **In-person**, in your hospital room, **or by phone**, if you have been discharged | Contact by the study coordinator and/or palliative care doctor to see how you are doing |  | X | 20 minutes |
| Day 7 | **In-person**, in your hospital room, **or by phone**, if you have been discharged | Contact by a different coordinator to collect a second set of questionnaires | X | X | 20 minutes |
| Day 30 | **In-person**, in your hospital room, **or by phone**, if you have been discharged | Contact by a different coordinator to collect the last set of questionnaires | X | X | 20 minutes |

## **Risks and Discomforts**

## **Emotional distress**: Talking about your illness and your symptoms may cause emotional distress for you or your caregiver. Our study coordinators will ask you the questions very gently and kindly. You may also get tired while answering the questions with the study coordinator. If you get too tired, we can come back later in the day. Sometimes people feel embarrassed or uncomfortable when being asked questions. You can refuse to answer any question.

## **Loss of confidentiality**: There is a chance that people not associated with the study will see your answers to questionnaires. Your name and other identifying information will be removed from study documents. All information (which we call data) will be kept in locked files in the study research offices at UAB. All the data will be housed in a secure, password-protected database at UAB.

## **Burden and benefits**: There is a risk related to being placed into a group by chance (like the flip of a coin). Patients in Group A may not have the same benefits as patients in Group B and patients in Group B may be more burdened by participating in the teleconsult visit with the palliative care doctor than patients in Group A.

## **Benefits**

There may be no direct benefits to you and others by participating in this study; however:

- **Palliative Care, Community and cultural training**: Participants in Group B may also experience a benefit from talking with a palliative care doctor who has been trained by the community members (who participated in developing the teleconsult program) in how to understand, talk to, and treat African American and white people in the rural South.
- **Future patients and caregivers**: Other people may benefit from your participation in this study (whether in Group A or Group B) in the future. This includes African American and white patients aged 65 and over and their caregivers in other parts of the country.

## **Alternatives**

The alternative is to not participate in the research.

**Confidentiality and Authorization to Use and Disclose Information for Research Purposes**

Federal regulations give you certain rights related to your health information. These include the right to know who will be able to get the information and why they may be able to get it. The study doctor must get your authorization (permission) to use or give out any health information that might identify you.

**What protected health information may be used and/or given to others?**

All medical information, including but not limited to information and/or records of any diagnosis or treatment of disease or condition, which may include sexually transmitted diseases (e.g., HIV, etc.) or communicable diseases, drug/alcohol dependency, etc.; all personal identifiers, including but not limited to your name, social security number, medical record number, date of birth, dates of service, etc.; any past, present, and future history, examinations, laboratory results, imaging studies and reports and treatments of any kind, including but not limited to drug/alcohol treatment, psychiatric/psychological treatment; financial/billing information, including but not limited to copies of your medical bills; any other information related to or collected for use in the research study, regardless of whether the information was collected for research or non-research (e.g., treatment) purposes; records about any study drug you received or about study devices used; and consent forms from past studies that might be in your medical record.

A description of this clinical trial will be available on [www.ClinicalTrials.gov](http://www.ClinicalTrials.gov), as required by U.S. Law. This website will not include information that can identify you. At most, the website will include a summary of the results. You can search this website at any time.

**Who may use and give out this information?**

Information about your health may be used and given to others by the study doctor and staff. They might see the research information during and after the study.

**Who might get this information?**

All individuals/entities listed in the informed consent document(s), including but not limited to, the physicians, nurses and staff and others performing services related to the research (whether at UAB or elsewhere). Your information may also be given to the sponsor of this research. “Sponsor” includes any persons or companies that are working for or with the sponsor, or are owned by the sponsor, or are providing support to the sponsor (e.g., contract research organization).

Information about you and your health which might identify you may be given to:

• The UAB Institutional Review Board (IRB). An IRB is a group that reviews the study to protect the rights and welfare of research participants.

• National Institute of Nursing Research/NIH/DHHS

• the Office for Human Research Protections (OHRP)

**Why will this information be used and/or given to others?**

Information about you and your health that might identify you may be given to others to carry out the research study. The sponsor will analyze and evaluate the results of the study. In addition, people from the sponsor and its consultants will be visiting the research site. They will follow how the study is done, and they will be reviewing your information for this purpose.

Information obtained during the course of the study which, in the opinion of the investigator(s), suggests that you may be at significant risk of harm to yourself or others will be reportable to a third party in the interest of protecting the rights and welfare of those at potential risk.

**What if I decide not to give permission to use and give out my health information?**

By signing this consent form, you are giving permission to use and give out the health information listed above for the purposes described above. If you refuse to give permission, you will not be able to be in this research.

**May I review or copy the information obtained from me or created about me?**

You have the right to review and copy your health information. However, if you decide to be in this study and sign this permission form, you will not be allowed to look at or copy your information until after the research is completed.

**May I withdraw or revoke (cancel) my permission?**

Yes, but this permission will not stop automatically. The use of your personal health information will continue until you cancel your permission.

You may withdraw or take away your permission to use and disclose your health information at any time. You do this by sending written notice to the study doctor. If you withdraw your permission, you will not be able to continue being in this study.

When you withdraw your permission, no new health information which might identify you will be gathered after that date. Information that has already been gathered may still be used and given to others. This would be done if it were necessary for the research to be reliable.

**Is my health information protected after it has been given to others?**

If you give permission to give your identifiable health information to a person or business, the information may no longer be protected. There is a risk that your information will be released to others. Including others outside of UAB, without your permission.

## **Voluntary Participation and Withdrawal**

Whether or not you take part in this study is your choice. There will be no penalty if you decide not to be in it. If you decide not to be in the study, you will not lose any benefits you are otherwise owed.

You are free to withdraw from this study at any time. Your choice to leave the study will not affect your relationship with this institution. However, you should return to see the study doctor for safety reasons so you can be taken off the study drug and referred for follow-up care. Contact the investigators Dr. Ronit Elk at 205-996-1702 or Dr. Marie Bakitas at 205-934-5277, or the overall study managers at 205-934-7905 (Emily Malone) or 205-975-0864 (Kristen Allen-Watts) if you want to withdraw from the study.

You may be removed from the study without your consent if the sponsor ends the study, if the study investigators decide it is not in the best interest of your health, or if you are not following the study rules.

## **Cost of Participation**

There will be no cost to you for taking part in this study.

## **Payment for Participation**

Regardless of whether you are in Group A or Group B, you will be paid $10 for completion of baseline questionnaires, $10 for completion of Day 7 questionnaires, and $20 for completion of the Day 30 questionnaire. The total payment you may receive is $40. If you do not finish the entire study, you will be paid at the time you decide to stop taking part in the study. This payment will be mailed to you as a check to the address you provide to the study coordinator.

## **New Findings**

You will be told by the study team if new information becomes available that might affect your choice to stay in the study.

## **Questions**

If you have any questions, concerns, or complaints about the research, please contact the principal investigators. You may contact Dr. Ronit Elk at 205-996-1702 or after hours 404-539-4827. You may also contact Dr. Marie Bakitas at 205-934-5277 or after hours at 603-398-7766.

If you have questions about your rights as a research participant, or concerns or complaints about the research, you may contact the UAB Office of the IRB (OIRB) at (205) 934-3789 or toll free at 1-855-860-3789. Regular hours for the OIRB are 8:00 a.m. to 5:00 p.m. CT, Monday through Friday.

## **Legal Rights**

You are not waiving any of your legal rights by signing this consent form.

## **Signatures**

Your signature below indicates that you have read (or been read) the information provided above and agree to participate in this study. You will receive a copy of this signed consent form.

Signature of Participant Date

Signature of Person Obtaining Consent Date

***Optional – Permission to record teleconsult visit***

Additionally, if you are assigned to receive the palliative care teleconsult visit, you understand that some visits may be audio recorded for quality control. Your signature below indicates that you agree to the teleconsult visit being audio recorded.

______________________________________________________________________________

Signature of Participant Date

**CAREGIVER CONSENT FORM**

**Title of Research:** A Community-Developed, Culturally-Based Palliative Care Tele-Consult Program for African American and White Rural Southern Elders with a Life Limiting Illness

**UAB IRB Protocol #:** IRB-300002420

**Principal Investigator:** Ronit Elk, Ph.D., and Marie Bakitas, DNSc

**Sponsor:** National Institute of Nursing Research/NIH/DHHS

| **General Information** | You and the person that you are caring for are being asked to take part in a research study. This research study is voluntary, meaning you do not have to take part in it. The procedures, risks, and benefits are fully described further in the consent form. |
| --- | --- |
| **Purpose** | The purpose of the study is to determine if palliative care delivered by a teleconsult visit is better than usual hospital care, without a palliative care teleconsult visit, in improving quality of life and management of symptoms, and reducing caregiver burden, for patients aged 65 and over in the rural South who are admitted to the hospital. There are no palliative care doctors in your area, but the teleconsult visit allows us to bring this special type of care to patients who would otherwise not be able to get it. ***Palliative care*** is a medical specialty that is concerned with improving patient’s quality of life and symptoms. Palliative care doctors work with the patient’s other medical doctors and health professionals to provide patients and families with an extra layer of support while they are dealing with a chronic illness. A ***teleconsult*** visit provides a typical evaluation by a specialist doctor; however, instead of the doctor visiting your loved one in his or her hospital room in-person, we will use a computer screen (like a tablet) to allow your loved one and the doctor to see each other through the screen (like Skype or FaceTime, except our video has special security to protect your privacy). |
| **Duration & Visits** | As the caregiver for a patient participating in this study, you will be asked to complete questionnaires at 3 time points:  • On the day of enrollment (Day 0): 30 minutes  • Day 7: 20 minutes  • Day 30: 20 minutes |
| **Overview of Procedures** | If the person that you are caring for is eligible for the study, you and the person that you are caring for will be approached, at the hospital, by a local community member who will introduce you to the study. If you agree to participate in the study, a coordinator will ask that you read this document and sign at the end. The coordinator will then ask that you complete baseline questionnaires. After you complete these baseline questionnaires, the person that you are caring for will be randomized (placed into a group by chance, like a flip of a coin) to either the usual care group or the palliative care intervention group.  The usual care group will receive medical care and supportive services that would be provided to any hospital patient. While the intervention group will receive the same care that is provided to any hospital patient in addition to receiving a teleconsult visit from a palliative care doctor.  Patients in the usual care group will receive medical care and supportive services that are provided to any hospital patient. Patients in the intervention group will receive the same care that is provided to any hospital patient in addition to receiving a teleconsult visit from a palliative care doctor. As the patient’s caregiver you are invited to participate in the teleconsult and in any follow up visits/calls so that you are aware of the palliative care services that are being provided and also available to you as a caregiver. On Day 3 and Day 6 after the teleconsult visit if the patient is still in the hospital, the coordinator and/or palliative care doctor will contact them in person or by phone to check on how they are doing and be sure that they have the resources they need when they leave the hospital. If your loved one is discharged from the hospital on Day 3 or Day 6 the coordinator and/or doctor will contact you by telephone using the contact information the patient will provide before they are discharged.  On Day 7 and Day 30, a different coordinator from the one that helped with the teleconsult will follow up to collect data by asking you questions about how your health and how satisfied you were with the care your loved one received in the hospital. These questions are the same or similar to the ones you were asked upon study enrollment. |
| **Risks** | This study has minimal risks. Some participants may feel anxious or bothered by the questionnaires. We have made these questionnaires as brief and simple as possible. If you don’t feel comfortable answering a question, you may leave it blank. Another risk is loss of confidentiality but we have protections in place to reduce the possibility of this happening. |
| **Benefits** | You may or may not directly benefit from taking part in this study. However, this study may help us better understand how to care for African American and white patients, with chronic illness, who are 65 years and older and their caregivers in the rural South. |
| **Alternatives** | The alternative is to not participate in this study. |

## **Purpose of the Research Study**

You and the person that you are caring for are invited to participate in a research study. Taking part in research is voluntary. The person that you are caring for will continue to receive the care prescribed by his or her doctors at the hospital whether he or she chooses to participate or not. Participation in this research program will be in addition to that care. This form explains what you will be asked to do if you and the person you are caring for decide to participate in this study. This form can be read aloud if you prefer. If you decide to participate in this study, you will be asked to sign this form. We will give you a signed copy of the form to keep. Please ask questions if there is anything you do not understand.

The purpose of the study is to learn how to improve quality of life and management of symptoms, and reduce caregiver burden, for patients aged 65 and over in the rural South who are admitted to the hospital through a teleconsult care visit with a palliative care doctor. The hospital doctor will remain in charge of the care for the patient and will take the palliative care doctor’s recommendation into consideration in providing his or her care.

The goal of this study is to see if receiving this teleconsult by a palliative care doctor, in addition to the care provided by your loved one’s doctors and nurses at this hospital, improves patient symptoms. There will be 352 patients (and their caregivers) across 3 hospitals (Alabama, South Carolina, and Mississippi) who will participate in the study. Half of the participants in this study (Group A) will receive the care that doctors and nurses at this hospital will provide for your loved one. Half of the participants in the study (Group B) will receive the palliative care program that’s been developed for patients in the rural South using a teleconsult, ***in addition*** to the care the doctors and nurses in this hospital provide.

## **Study Participation & Procedures**

If you agree to join the study, you and the person you are caring for will go through the following steps:

1. **Consent Form**: If you and the person you are caring for agree to enroll in this research study, you will be asked to sign this consent form.
2. **Questionnaires**: The study coordinator will ask you, the caregiver, to answer some questions about how you feel, how things are going for you, and what it’s like to care for someone with a chronic illness. This will take about 40 minutes.
3. **Group Assignment (*patient participant only*)**: After you and the patient participant have completed the first set of questionnaires with the study coordinator, the person that you are caring for will be assigned by chance (like the flip of a coin) to one of the two study groups (either Group A or Group B). No one can control which group you will be assigned, not the doctor, the study coordinator, the nurse or you; it is done by a computer program. Once the patient is assigned to a group, one of the study coordinators who will be responsible for collecting questionnaire data on Days 7 and 30 will not know which group you have been assigned to. This is called blinding. You will be asked not to tell the coordinator which group the patient participant has been assigned to.

**If the *person you are caring* for is assigned to Group A** – ***Usual medical care***:

- **Medical Care**: Patients in Group A receive the medical care and supportive services that would be provided to any hospital patient.
- **Questionnaires**: You will be asked to answer some questions about your health, what it’s like to care for someone with a chronic illness, and how satisfied you are with the patient participant’s care at baseline (Day 0) (questionnaires will take approximately 40 minutes), Day 7 (questionnaires will take approximately 20 minutes), and Day 30. The study coordinator will also contact you by phone to ask a few questions (questionnaire will take approximately 20-25 minutes).

If the patient participant should pass away during the study, we would like to contact you to ask some additional questions about their care. The study coordinator will contact you by phone to ask a few questions (questionnaires will take approximately 20-25 minutes).

**If the *person you are caring* for is assigned to Group B – *Usual medical care AND teleconsult visit***:

- **Medical Care**: Patients in Group B receive the medical care and supportive services that would be provided to any hospital patient and the palliative care teleconsult visit.

If the patient participant is in this group, a study coordinator will schedule a meeting for him or her with the palliative care doctor, at a time that is convenient for you and your loved one either later today or tomorrow. For the appointment, the study coordinator will bring in a special screen (computer or tablet) and introduce you and the person you are caring for to the palliative care doctor (the study provides all of this equipment – you will not need to have a computer, tablet, or smartphone to participate). You will be able to see and hear the doctor and she or he will be able to see and hear you. The palliative care teleconsult visit will take place in your loved one’s hospital room. The study coordinator will stay in the room with you to make sure the screen is working well and provide assistance. The appointment with the palliative care doctor may take up to 1 hour. At the end of the appointment the palliative care doctor will discuss their recommendations for treatment with you and your loved one.

**Please note some of the teleconsult visits will be audio recorded using a secure device for quality reasons**. We will only be audio recording the teleconsult visit conversation. The recording will only be reviewed by a trained member of the study team to make sure the palliative care doctor is covering all of the important points during the visit. After all study activities and data analysis are completed, all recordings will be destroyed.

- **Report to the patient participant’s doctor**: Shortly after the teleconsult visit, the palliative care doctor will put a note, of his or her recommendations that he or she discussed with you and the patient, into the patient’s electronic medical chart at the hospital for the hospital care team to see. He or she may also call your hospital doctor to talk through the recommendations.
- **Contact #2 Day 3** (approximately 72 hours after the teleconsult visit with the study coordinator who was involved in the visit and/or the palliative care doctor; by video if your loved one is in hospital, by phone if he or she has been discharged.) The purpose of the second contact is to determine how the patient is feeling, if the palliative care doctor’s plan was implemented, if you have encountered any challenges or questions, and to determine who will be assuming your loved one’s care once they leave the hospital.
- **Contact #3 Day 6** (approximately 6 days after the teleconsult visit with the palliative care doctor; by video if your loved one is in hospital, by phone if he or she has been discharged.) The purpose of the third contact is to determine if the patient is experiencing new or ongoing challenges and to confirm availability of the care they need when they leave the hospital.
- **Questionnaires**: On the first day (Day 0) of the study, you will be asked to answer some questions about your quality of life, how you’re feeling, and how satisfied you are with the care your loved one received while in the hospital at Day 0 (these baseline questionnaires will take approximately 30 minutes), on Day 7 (questionnaires will take approximately 20 minutes) and on Day 30 (questionnaires will take approximately 20 minutes).

If the patient participant should pass away during the study, we would like to contact you to ask some additional questions about their care. The study coordinator will contact you by phone to ask a few questions (questionnaires will take approximately 20 minutes).

| **TIMELINE** | | | | | |
| --- | --- | --- | --- | --- | --- |
| **When** | **Where** | **What** | **Group A** | **Group B** | **Approx.**  **Duration** |
| Today (Day 0) | **In-person**, in your loved one’s hospital room | 1. Review and sign consent form  2. Complete first set of questionnaires | X | X | 1. Consent: 30 min  2. Questionnaires: 30 min  Total: up to 1 hour |
| Today or tomorrow | **In-person**, in your loved one’s hospital room | Teleconsult appointment with the patient participant and a palliative care doctor |  | X | 45-60 minutes |
| Day 3 | **In-person**, if your loved one is still in the hospital, **or by phone**, if he or she has been discharged | Contact by a study coordinator and/or palliative care doctor to see how you are doing |  | X | 20 minutes |
| Day 6 | **In-person**, if your loved one is still in the hospital, **or by phone**, if he or she has been discharged | Contact by a study coordinator and/or palliative care doctor to see how the patient participant is doing and to be sure he or she has everything he or she needs at home. |  | X | 20 minutes |
| Day 7 | **In-person**, if your loved one is still in the hospital, **or by phone**, if he or she has been discharged | Second set of questionnaires | X | X | 20 minutes |
| Day 30 | **In-person**, if your loved one is still in the hospital, **or by phone**, if he or she has been discharged | Last set of questionnaires | X | X | 20 minutes |

## **Risks and Discomforts**

- **Emotional distress**: Talking about illness and symptoms may cause emotional distress for you, the caregiver. Our study coordinators will ask you the questions very gently and kindly. You may also get tired while answering the questions with the study coordinator. If you get too tired, we can come back later in the day. Sometimes people feel embarrassed or uncomfortable when being asked questions. You can refuse to answer any question.
- **Loss of confidentiality**: There is a chance that people not associated with the study will see your answers to questionnaires. Your name and other identifying information will be removed from study documents. All information (which we call data) will be kept in locked files in the study research offices at UAB. All the data will be housed in a secure, password-protected database at UAB.

## **Benefits**

There may be no direct benefits for you by participating in this study. However, this study may help us better understand how to care for African American and white patients who are 65 years and older, and their caregivers, in the rural South.

## **Alternatives**

The alternative is to not participate in the research.

## **Confidentiality**

Information obtained about you for this study will be kept confidential to the extent allowed by law. However, research information that identifies you may be shared with people or organizations for quality assurance or data analysis, or with those responsible for ensuring compliance with laws and regulations related to research. They include:

- the UAB Institutional Review Board (IRB). An IRB is a group that reviews the study to protect the rights and welfare of research participants.
- the National Institute of Nursing Research/NIH/DHHS
- the Office for Human Research Protections (OHRP)

The information from the research may be published for scientific purposes; however, your identity will not be given out in those publications.

A description of this clinical trial will be available on [www.ClinicalTrials.gov](http://www.ClinicalTrials.gov), as required by U.S. Law. This website will not include information that can identify you. At most, the website will include a summary of the results. You can search this website at any time.

Information obtained during the course of the study which, in the opinion of the investigator(s), suggests that you may be at significant risk of harm to yourself or others will be reportable to a third party in the interest of protecting the rights and welfare of those at potential risk.

## **Voluntary Participation and Withdrawal**

Whether or not you take part in this study is your choice. There will be no penalty if you decide not to be in it. If you decide not to be in the study, you will not lose any benefits you are otherwise owed.

You are free to withdraw from this study at any time. Your choice to leave the study will not affect your relationship with this institution. Contact the investigators Dr. Ronit Elk at 205-996-1702 or Dr. Marie Bakitas at 205-934-5277, or the overall study managers at 205-934-7905 (Emily Malone) or 205-975-0864 (Kristen Allen-Watts), if you want to withdraw from the study.

You may be removed from the study without your consent if the sponsor ends the study, if the study drug is approved by the FDA, if the study doctor decides it is not in the best interest of your health, or if you are not following the study rules.

## **Cost of Participation**

There will be no cost to you for taking part in this study.

## **Payment for Participation**

You will be paid $10 for completion of the Day 0 questionnaires, $10 for completion of Day 7 questionnaires, and $20 for completion of the Day 30 questionnaires. The total payment you may receive is $40. If you do not finish the entire study, you will be paid at the time you decide to stop taking part in the study. This payment will be mailed to you in the form of a check at the address you provide to the study coordinator.

## **New Findings**

You will be told by the study team if new information becomes available that might affect your choice to stay in the study.

## **Questions**

If you have any questions, concerns, or complaints about the research, please contact the principal investigators. You may contact Dr. Ronit Elk at 205-996-1702 or after hours 404-539-4827. You may also contact Dr. Marie Bakitas at 205-934-5277 or after hours at 603-398-7766.

If you have questions about your rights as a research participant, or concerns or complaints about the research, you may contact the UAB Office of the IRB (OIRB) at (205) 934-3789 or toll free at 1-855-860-3789. Regular hours for the OIRB are 8:00 a.m. to 5:00 p.m. CT, Monday through Friday.

## **Legal Rights**

You are not waiving any of your legal rights by signing this consent form.

## **Signatures**

Your signature below indicates that you have read (or been read) the information provided above and agree to participate in this study. You will receive a copy of this signed consent form.

Signature of Caregiver Participant Date

Signature of Person Obtaining Consent Date

***Optional – Permission to record teleconsult visit***

Additionally, if the person you are caring for is assigned to receive the palliative care teleconsult visit, you understand that some visits may be audio recorded for quality control. Your signature below indicates that you agree to the teleconsult visit being audio recorded.

________________________________________________________________________

Signature of Caregiver Participant Date
